# A reappraisal of the prevalence of pediatric hypertension through a nationwide database in Taiwan

**DOI:** 10.1038/s41598-021-84001-6

**Published:** 2021-02-24

**Authors:** Wan-Fu Hsu, Yi-Wei Kao, Mingchih Chen, Huei-Chen Chiang, Shih-Yen Chen, Meng-Che Lu, Ben-Chang Shia, Kai-Sheng Hsieh

**Affiliations:** 1 Department of Pediatrics, Tri-Service General Hospital, National Defense Medical Center, Taipei, Taiwan, ROC; 2 Graduate Institute of Business Administration, College of Management, Fu Jen Catholic University, New Taipei City, Taiwan, ROC; 3 Research Center of Big Data, College of Management, Taipei Medical University, Taipei, Taiwan, ROC; 4 Department of Pediatrics, Shuangho Hospital, Taipei Medical University, New Taipei City, Taiwan, ROC; 5 Taipei Heart Institute, Taipei Medical University, Taipei, Taiwan, ROC

**Keywords:** Hypertension, Epidemiology, Paediatric research

## Abstract

Hypertension in childhood and adolescence is associated with adult cardiovascular morbidity and mortality. However, the reported prevalence of pediatric hypertension varies considerably. We conducted a pioneer nationwide population-based study to investigate the prevalence of hypertension among children and adolescents. Pediatric patients who had been diagnosed with hypertension between 2000 and 2013 were selected from the National Health Insurance Research Database in Taiwan. Other metabolic syndrome-related diseases that would increase cardiovascular risk, including diabetes mellitus (DM), hyperlipidemia, and obesity, were also retrieved for further evaluation. In total, 10,364 children and adolescents diagnosed with hypertension were identified. The prevalence of pediatric hypertension in Taiwan ranged from 0.19 to 0.38 per 1000 children and adolescents between 2000 and 2013. Essential hypertension was most commonly coded (90.6%), which was much more than secondary hypertension (14.3%). Children and adolescents with hypertension were often associated with DM, hyperlipidemia, and obesity, with the odds ratios as 14.05 (95% confidence interval (CI) 11.74–16.81, p < 0.001), 10.65 (95% CI 9.48–11.97, p < 0.001), and 19.08 (95% CI 15.65–23.26, p < 0.001), respectively. To improve lifelong cardiovascular health, our results emphasize the importance of early proper recognition and suitable management of hypertension, as well as metabolic syndrome-related diseases, among children and adolescents.

## Introduction

Hypertension is recognized as a major risk factor for morbidity and mortality in adulthood^1,2^. Nevertheless, the prevalence of adult hypertension is still increasing; one recent systematic review revealed that the prevalence of hypertension in adults had increased from 25.9% in 2000 to 31.1% in 2010^3^. Some evidence has demonstrated that adult hypertension originates in childhood^4,5^. On the other hand, children with elevated blood pressure (BP) are found to be at risk of developing hypertension in adulthood^6,7^. The presence of hypertension during early life was also found to be associated with metabolic syndrome^6^, thickening of the carotid intima-media^8,9^, increased left ventricular mass and arterial stiffness^9^, and impaired renal^10^, cardiac diastolic^9^, and neurocognitive functions^11^. Therefore, BP control in early life is important and meaningful.

The reported prevalence of hypertension in children and adolescents were widely ranged. In Asia, South-Asian studies showed the prevalence of hypertension in children and adolescents to be 26.34% in Pakistan^12^, 7 ~ 21.5% in India^13,14^, and 24.5% in Malaysia^15^. Data from East Asia demonstrated relatively lower prevalence; pediatric hypertension was estimated to be 10.6% in China^16^, 1.9 ~ 4.4% in Korea^17^, and 0.1 ~ 3% in Japan^18^. Because multiple factors are related to BP, such as lifestyle, socioeconomic status, personal medical history, inheritance, ethnicity, etc.^16,19,20^, different prevalence of hypertension among children and adolescents from different area was reasonable; these findings also enhanced the significance to understand the prevalence of pediatric hypertension among different populations. Besides, since most of the above studies were health screenings for specific groups, to reflect the conditions of entire populations, we believe a nationwide research will be more representative.

Diabetes mellitus (DM), hyperlipidemia, and obesity are known to be associated with pediatric hypertension^19,20^; additionally, these diseases are also risk factors of cardiovascular morbidity and mortality. Therefore, to investigate the associated risk of these metabolic syndrome-related diseases among pediatric patients with hypertension, was another focus of the present study.

In Taiwan, the National Health Insurance (NHI) program was implemented in 1995. It provides healthcare for up to 99.99% of the population (more than 23 million people). The National Health Insurance Research Database (NHIRD), which uses International Classification of Diseases, Ninth Revision, Clinical Modification (ICD-9-CM) codes to register diagnoses, contains all claims data of beneficiaries in the NHI program. The clinical application of the database was validated from several studies, and the sensitivity and specificity were 0.75 and 0.93 for hypertension^21,22^. As a result, the medical database of the NHI program can illuminate the disease burden and healthcare status of the entire population of Taiwan. To investigate the prevalence of hypertension and the associated risk of metabolic syndrome-related diseases among children and adolescents in Taiwan, we conducted a pioneer nationwide population-based study using the NHIRD.

## Results

### The prevalence of hypertension among children and adolescents

**Figure 1:**
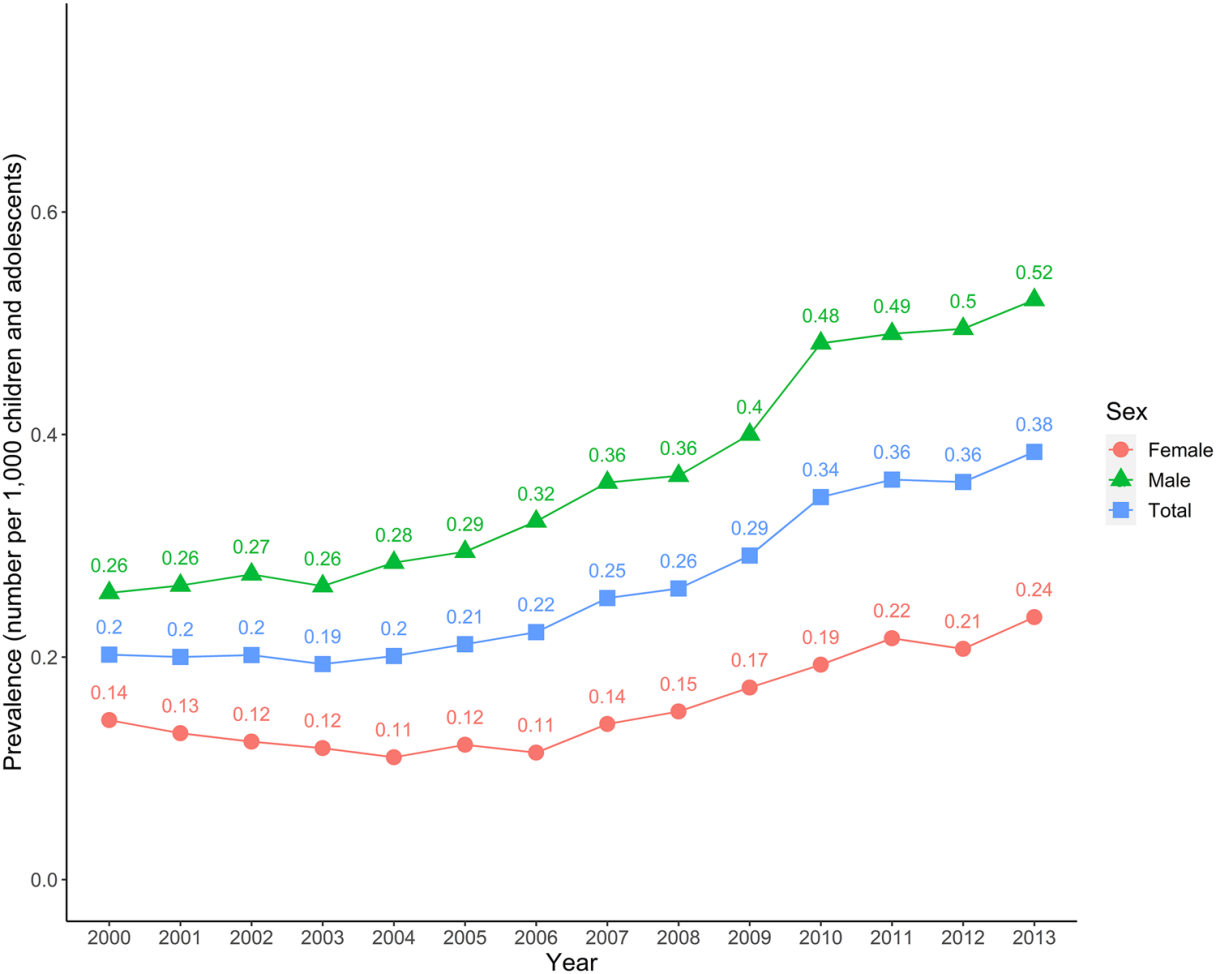
The annual prevalence of hypertension among children and adolescents and its trends from 2000 to 2013. The figure was generated by R Statistical Software, vers. 3.6.1. (R Foundation for Statistical Computing, Vienna, Austria; http://www.r-project.org).

In total, 10,364 (71.1% male) hypertensive children and adolescents were identified. The yearly prevalence of hypertension in the pediatric population ranged from 0.19 to 0.38 per 1000 children and adolescents between 2000 and 2013. The mean age of the hypertensive patients was 13.16 ± 4.43 years. The population of overall children and adolescents and those with hypertension in each year from 2000 to 2013 was demonstrated in Table 1. The trend in annual prevalence of pediatric hypertension during the study period was demonstrated in Fig. 1.

**Table 1:** The population of overall children and adolescents and of those with hypertension in each year from 2000 to 2013.

Number	Overall population of children and adolescents of each year			Population of children and adolescents with hypertension of each year		
Year	Female	Male	All	Female	Male	All
2000	2,894,726	3,074,053	5,968,779	415	792	1207
2001	2,836,221	3,030,166	5,866,387	373	801	1174
2002	2,767,066	2,970,860	5,737,926	343	815	1158
2003	2,699,903	2,911,965	5,611,868	319	768	1087
2004	2,637,755	2,857,168	5,494,923	290	814	1104
2005	2,592,200	2,816,572	5,408,772	314	830	1144
2006	2,541,290	2,764,748	5,306,038	290	890	1180
2007	2,474,820	2,695,686	5,170,506	346	962	1308
2008	2,423,443	2,641,765	5,065,208	366	959	1325
2009	2,358,137	2,569,928	4,928,065	407	1028	1435
2010	2,299,391	2,503,980	4,803,371	444	1207	1651
2011	2,226,777	2,423,800	4,650,577	483	1189	1672
2012	2,165,233	2,357,366	4,522,599	449	1167	1616
2013	2,124,112	2,310,995	4,435,107	501	1204	1705

### Association between hypertension and metabolic syndrome-related diseases among children and adolescents

Among the identified hypertensive children and adolescents, 4747 (45.8%) were also coded with metabolic syndrome-related diseases, including 1829 (17.6%) with DM, 3472 (33.5%) with hyperlipidemia, and 1990 (19.2%) with obesity. Most identified DM patients were coded with type 2 DM and some patients were doubly coded during the study period. The risks of metabolic syndrome-related diseases, including DM, hyperlipidemia, and obesity, were significantly higher in the hypertension group than the non-hypertension control group, with the ORs as 14.05 (95% CI 11.74–16.81, *p* < 0.001), 10.65 (95% CI 9.48–11.97, *p* < 0.001), and 19.08 (95% CI 15.65–23.26, *p* < 0.001), respectively. The paired comparison of metabolic syndrome-related diseases in each group is summarized in Table 2.

**Table 2:** Paired comparison of metabolic syndrome-related diseases in children and adolescents with hypertension and non-hypertension.

Number (%)	Hypertension	Non-Hypertension	OR (95% CI)
	10,364	10,364	
**Sex**			
Male	7371 (71.1%)	7371 (71.1%)	
Female	2993 (28.9%)	2993 (28.9%)	
Age (years)	13.16 ± 4.43	13.16 ± 4.43	
**Age groups (years)**			
0–6	1259 (12.1%)	1259 (12.1%)	
6–12	1646 (15.9%)	1646 (15.9%)	
12–18	7459 (72.0%)	7459 (72.0%)	
**Metabolic syndrome-related diseases**			
**Diabetes mellitus**	1829 (17.6%)	159 (1.5%)	14.05 (11.74–16.81)*
Type 1 DM	280 (2.7%)	15 (0.1%)	19.93 (11.65–34.09)*
Type 2 DM	1772 (17.1%)	149 (1.4%)	14.2 (11.82–17.04)*
DM without mention of type	22 (0.2%)	3 (0.0%)	7.33 (2.2–24.5)*
Hyperlipidemia	3472 (33.5%)	499 (4.8%)	10.65 (9.48–11.97)*
Obesity	1990 (19.2%)	128 (1.2%)	19.08 (15.65–23.26)*
DM or hyperlipidemia	3996 (38.6%)	585 (5.6%)	11.34 (10.13–12.69)*
DM and hyperlipidemia	1305 (12.6%)	73 (0.7%)	18.59 (15.91–21.72)*
DM or obesity	3205 (30.9%)	268 (2.6%)	13.14 (11.71–14.75)*
DM and obesity	614 (5.9%)	19 (0.2%)	13.67 (12.2–15.3)*
Hyperlipidemia or obesity	4371 (42.2%)	595 (5.7%)	20.87 (16.18–26.93)*
Hyperlipidemia and obesity	1091 (10.5%)	32 (0.3%)	34.06 (21.31–54.42)*
DM or hyperlipidemia or obesity	4747 (45.8%)	669 (6.5%)	35.16 (24.61–50.25)*
DM and hyperlipidemia and obesity	466 (4.5%)	7 (0.1%)	66.57 (31.56–140.42)*

*CI* confidence interval, *DM* diabetes mellitus, *OR* odds ratio, *SD* standard deviation.

*p < 0.001.

### Distribution in diagnostic codes among pediatric hypertension

The distribution in diagnostic codes among pediatric hypertension according to ICD-9-CM are demonstrated in Table 3. Essential hypertension was most commonly coded among hypertensive children and adolescents (90.6%), followed by hypertensive heart disease (21.5%), secondary hypertension (14.3%), hypertensive chronic kidney disease (CKD) (7.2%), and hypertensive heart and CKD (2.4%).

**Table 3:** Distribution in diagnostic codes of hypertension among children and adolescents according to ICD-9-CM.

Number (%)	Overall	Male	Female	*p*
	10,364	7371	2993	
Essential hypertension	9387 (90.6%)	6756 (91.7%)	2631 (87.9%)	< 0.001
Hypertensive heart disease	2233 (21.5%)	1636 (22.2%)	597 (19.9%)	0.013
Hypertensive CKD	751 (7.2%)	432 (5.9%)	319 (10.7%)	< 0.001
Hypertensive heart and CKD	245 (2.4%)	165 (2.2%)	80 (2.7%)	0.212
Secondary hypertension	1484 (14.3%)	1055 (14.3%)	429 (14.3%)	1.000

*CKD* chronic kidney disease, *ICD-9-CM* International Classification of Diseases, Ninth Revision, Clinical Modification.

An analysis of the distribution in subcodes of each hypertension, based on the classification of ICD-9-CM, is demonstrated in Table 4. To protect personal information, the NHIRD was only available for on-site analysis after 2016^21^; additionally, if the case number was fewer than three, it may increase the risk of personal information being identified and the data were not allowed to be taken out. Thus, some subtypes with fewer than three cases are not presented.

**Table 4:** Distribution in subcodes of each hypertension among children and adolescents according to ICD-9-CM.

Number (%)	Overall	Male	Female	*p*
	10,364	7,371	2,993	
**Essential hypertension**	9387 (90.6%)	6756 (91.7%)	2631 (87.9%)	< 0.001
Malignant essential hypertension	370 (3.6%)	255 (3.5%)	115 (3.8%)	0.372
Benign essential hypertension	1894 (18.3%)	1442 (19.6%)	452 (15.1%)	< 0.001
Unspecified essential hypertension	8199 (79.1%)	5991 (81.3%)	2208 (73.8%)	< 0.001
**Hypertensive heart disease**	2233 (21.5%)	1636 (22.2%)	597 (19.9%)	0.013
Malignant hypertensive heart disease	126 (1.2%)	88 (1.2%)	38 (1.3%)	0.826
Benign hypertensive heart disease	613 (5.9%)	482 (6.5%)	131 (4.4%)	< 0.001
Unspecified hypertensive heart disease	1664 (16.1%)	1214 (16.5%)	450 (15.0%)	0.076
**Hypertensive CKD**	751 (7.2%)	432 (5.9%)	319 (10.7%)	< 0.001
Malignant hypertensive renal disease	67 (0.6%)	49 (0.7%)	18 (0.6%)	0.818
Benign hypertensive renal disease	106 (1.0%)	61 (0.8%)	45 (1.5%)	0.003
Unspecified hypertensive renal disease	586 (5.7%)	338 (4.6%)	248 (8.3%)	< 0.001
**Hypertensive heart and CKD**	245 (2.4%)	165 (2.2%)	80 (2.7%)	0.212
Malignant hypertensive heart and renal disease	21 (0.2%)	14 (0.2%)	7 (0.2%)	0.834
Benign hypertensive heart and renal disease	49 (0.5%)	35 (0.5%)	14 (0.5%)	1.000
Unspecified hypertensive heart and renal disease	176 (1.7%)	118 (1.6%)	58 (1.9%)	0.263
**Secondary hypertension**	1484 (14.3%)	1055 (14.3%)	429 (14.3%)	1.000
Malignant secondary hypertension	103 (1.0%)	71 (1.0%)	32 (1.1%)	0.701
Benign secondary hypertension	239 (2.3%)	172 (2.3%)	67 (2.2%)	0.826
Unspecified secondary hypertension	1190 (11.5%)	843 (11.4%)	347 (11.6%)	0.847

*CKD* chronic kidney disease, *ICD-9-CM* International Classification of Diseases, Ninth Revision, Clinical Modification.

Among the subcodes of essential hypertension, unspecified essential hypertension was most commonly coded. The proportion of overall essential hypertension in male children and adolescents was significantly higher than that in females (*p* < 0.001); the same conditions were also seen in benign essential hypertension and unspecified essential hypertension (*p* < 0.001 each).

The major subcode of hypertensive heart disease was unspecified hypertensive heart disease. The proportion of overall hypertensive heart disease in male children and adolescents was significantly higher than that in females (*p* < 0.05); the same condition was also seen in benign hypertensive heart disease (*p* < 0.001).

In contrast to essential hypertension and hypertensive heart disease, the proportion of overall hypertensive CKD in female children and adolescents was significantly higher than that in males (*p* < 0.001); the same conditions were also seen in benign hypertensive renal disease and unspecified hypertensive renal disease (*p* < 0.05 each). Among the subcodes of hypertensive CKD, the most common coded diagnosis was unspecified hypertensive renal disease.

The most common subcode among hypertensive heart and CKD was unspecified hypertensive heart and renal disease. Proportions of each subcode of hypertensive heart and CKD between males and females did not significantly differ.

Proportions of each subcode of secondary hypertension did not significantly differ between males and females. Among the subcodes of secondary hypertension, unspecified secondary hypertension was most common.

## Discussion

This is the first nationwide population-based study to explore the prevalence of hypertension and the associated risk of metabolic syndrome-related diseases among children and adolescents. Additionally, instead of conducting a health survey, which relies on a physiological measurement of blood pressure, hypertensive children and adolescents in our study were identified from the NHIRD; we believe such a research approach is more representative of the current situation of the entire pediatric group in Taiwan. There are several important points that can be drawn from the study. First, the prevalence of pediatric hypertension in Taiwan ranged from 0.19 to 0.38 per 1000 children and adolescents and it was increasing gradually during the entire study period. Second, in comparison to the non-hypertension group, children and adolescents with hypertension were found to be at higher risks for metabolic syndrome-related diseases, including DM, hyperlipidemia, and obesity. Third, according to ICD-9-CM, the most common code of hypertension among children and adolescents was essential hypertension. Forth, the proportion of malignant hypertension among each code of hypertension was very small; further studies are undertaken to investigate the long-term outcome and the association between pediatric and adult malignant hypertension. Although the prevalence of pediatric hypertension was not high in Taiwan, the present study did reveal the medical claims by the physicians; additionally, it may also reflect the current state of healthcare providers' awareness of these issues.

Among the study population, there were 3472 (33.5%) pediatric patients with both hypertension and hyperlipidemia. In the past, lipid measurements were not frequently conducted in pediatrics^23^. However, dyslipidemia in children and adolescents was found to be linked with several comorbidities, including being a predictor of thickening of the carotid intima-media^23,24^, which is one of the risk factors for future cardiovascular events^25^. Therefore, lipid profiles should be carefully monitored in children and adolescents, especially in pediatric patients with other cardiovascular risks, such as hypertension. Once dyslipidemia is found, management of these children includes lifestyle modifications, such as weight loss, diet control, exercise, etc.; pharmacology for lowering lipid levels can be used in some conditions^26,27^.

With the continuous increase in prevalence, obesity was proposed as being a global health challenge^28^. Recent studies showed that the prevalence of hypertension is higher in obese children and adolescents^29–31^. Nevertheless, there are also studies which found that a correlation between secular changes in hypertension and obesity in children was not very strong^17,32^. Such results may reflect conclusions by Cheung et al., although obesity is recognized as a strong predictor of early hypertension, the association still varies among different ethnicities^33^. In our study, the occurrence of obesity in hypertensive children and adolescents was 19.2%, which was significantly higher than that of the non-hypertension group. However, because the information regarding body-mass index (BMI) was unavailable in the database, the patients with obesity were identified based on the claims data in NHIRD, instead of BMI. Therefore, we could not further investigate the association between pediatric hypertension and BMI. Further study is undertaken to evaluate the relationship between hypertension and obesity among children and adolescents in Taiwan.

Both type 1 and type 2 DM are known to be associated with hypertension in children and adolescents; additionally, the rate of developing hypertension was higher in patients with type 2 DM than those with type 1 DM^34,35^. In our study, we also found that children and adolescents with hypertension were associated with higher risks of DM than those without hypertension, regardless of the type of DM. Nevertheless, inadequate awareness of hypertension and unsatisfactory outcomes of anti-hypertensive treatment among children and adolescents with either type 1 or type 2 DM were reported^34,36^. Further analysis revealed type 2 DM was predominant in our study group; the result was compatible with the findings by Zeitler et al., they noted the prevalence of youth-onset type 2 DM was much higher in non-Caucasian descendants^37^. Therefore, our findings emphasized again that children and adolescents with DM need to closely monitor their BP, similarly, children and adolescents with hypertension should also closely monitor their blood glucose. We believe the recommendation were also applicable to pediatric patients with hyperlipidemia and obesity to improve their cardiovascular health.

In the past, secondary hypertension was generally thought to be more common in children and adolescents than in adults^38^; however, in some more-recent studies on pediatric hypertension, patients with primary hypertension were more than secondary hypertension^39,40^. Similarly, the present study also found that primary hypertension among children and adolescents in Taiwan was more common than secondary hypertension. The analysis by age groups also revealed that the age group 12–18 years old accounted for the largest proportion and followed by the age group 6–12 years old. The results revealed that, among the children and adolescents with hypertension in Taiwan, more than 87% of these patients were diagnosed after the age of 6 years. Because relatively older age (more than 6 years old) is one of the general characteristics of children with primary hypertension, extensive assessment of other secondary causes could be reserved for younger children (0–6 years old) and those with other risk factors^19^. Once children and adolescents were diagnosed with hypertension, non-pharmacologic intervention such as lifestyle modification are still preferred initially; however, in patients with advanced conditions or other secondary causes, pharmacologic therapy and specific intervention for underlying diseases should be considered^19^. Interestingly, although the association between pediatric hypertension and CKD is well-established and the reported prevalence of hypertension among children and adolescents with CKD could be as high as 54%^41–43^; our data revealed that the prevalence of hypertension among children and adolescents with CKD was only 9.6%. There are two possible reasons for this discrepancy: (1) the main cause of hypertension in our study group was essential hypertension; thus the ratio of other causes was decreased; (2) the result may imply that hypertension was not strongly correlated with renal diseases in Taiwanese children and adolescents, probably because pediatric patients with renal diseases can receive timely and suitable medical therapy via the easily accessible medical care system in Taiwan. Further studies are necessary to clarify this point.

Compared to reports from the published literatures, the rate of pediatric hypertension in Taiwan was relatively low^12–19,44^. Possible reasons for this might be multifactorial. First, according to the guidelines, both pediatric and adult patients, BP should be measured repeatedly on separate occasions to confirm the diagnosis of hypertension^19,20,45–47^. However, most of the reported prevalence were estimated based on the health survey in selected groups and the BP was measured with different intervals at the same visit. The present study was conducted through a nationwide database, in which the diagnoses were required to be confirmed by the attending physicians; additionally, we excluded children and adolescents who had an outpatient clinic visit under the diagnosis of hypertension less than twice to avoid including patients with tentative diagnoses. We believe this approach could avoid the limitation of a single health survey and would be more compatible with the definitions of current guidelines^19,20^. Second, if children and adolescents have a known primary disease, it would have been coded as the main diagnosis; however, if a patient was simultaneously found to have hypertension, the coding of hypertension might be missed. Frequent under-diagnosis of hypertension in pediatric patients was previously demonstrated^44,48^. Although the medical care system in Taiwan is efficient and easily accessible, because of a lack of early symptoms and signs in hypertension, children and adolescents with hypertension might not seek medical help. In addition, young children usually find it difficult to cooperate during BP measurements. Nevertheless, early and regular BP evaluation during each healthcare encounter in pediatric patients are strongly encouraged; this is also recommended by recent guidelines^19^. Third, there could be ethnicity/genetic factors in play. Cheung et al. performed a school-based BP screening in over 20,000 adolescents and demonstrated that although obesity is recognized as a strong predictor of the early development of hypertension, the strength of the association varies from race to race^33^. Fourth, there are other environmental factors. A national survey in China found that the prevalence of hypertension in children and adolescents from different regions significantly differed^16^. The authors postulated that the socioeconomic status, especially dietary habits, may have played an important role.

There are several significant changes in newly published guidelines for high BP in children and adolescents, including a new percentile reference, replacement of the term “prehypertension” with a new term “elevated BP”, a simplified screening table for recognizing abnormal BP, updated definitions of BP categories and stages, etc.^19^. Although hypertension and metabolic syndrome-related diseases are not major causes of death in children and adolescents^49,50^, as we mentioned earlier, adult hypertension is a leading cause of mortality^1^. Abundant evidence has demonstrated that adult hypertension originates in childhood^4,5^. As a result, to promote lifelong cardiovascular health, efforts to improve the diagnosis and management of pediatric hypertension are crucial.

The present study had several limitations. First, the research was based on the claims data recorded in the NHIRD, and the diagnoses relied only on ICD-9-CM codes. Therefore, some information was not available for evaluation, including the clinical severity of hypertension and metabolic syndrome-related diseases. Second, the diagnosis and coding of hypertension and metabolic syndrome-related diseases may have been inadequate because BP is rarely measured in children if there are no significant symptoms or signs. In addition, if pediatric patients seek medical care due to other known diseases, such as renal disease, inadequate coding of hypertension may be possible because it may be considered not significant. Nevertheless, we suppose the clinicians would be competent to make appropriate diagnoses based on the clinical practice guideline after adequate training and mandatory continued medical education. As a result, we believe the present study still offers a useful summary of hypertension and metabolic syndrome-related diseases among children and adolescents in Taiwan using our national health database. Additionally, the results may also remind healthcare providers to pay more attention to these issues. Third, we did not use the information of medications to improve the certainty of hypertension diagnosis because drug therapy in children and adolescents with hypertension were not the primary option^19,45^. Forth, owing to the limitation of the ICD-9-CM codes, it was possible if patients were noted to have cardiac problems, patients with primary or secondary hypertension would both be coded as hypertensive heart disease. This situation may also occur in patients coded as hypertensive CKD, and hypertensive heart and CKD. As a result, further research to clarify the natural history of these patients is warranted. Fifth, other factors, such as the BMI, genetic, occupation, stress events, and environmental factors, were not available for further investigation in the NHIRD. However, because more than 99% of the Taiwanese population participates in the NHI program, the data should be representative without institutional or cohort bias.

In conclusion, although the prevalence of hypertension among children and adolescents was low, its number was increasing. The patients who had an outpatient clinic visit under the diagnosis of hypertension less than twice would be excluded; we believed the approach will be more compatible with current guidelines. Most of the identified children and adolescents with hypertension were diagnosed with primary hypertension, rather than secondary hypertension. To the best of our knowledge, this is the first study to evaluate the prevalence and associated risks of metabolic syndrome-related disease among children and adolescents with hypertension through a nationwide database. We believe these findings can offer healthcare providers more-comprehensive insights into these issues and help establish better screening and therapeutic policies to further improve lifelong cardiovascular health.

## Methods

### Data sources and study population

Between January 1, 2000, and December 31, 2013, children and adolescents diagnosed with hypertension based on ICD-9-CM codes (401 ~ 405) were identified from the NHIRD. To avoid including children and adolescents with tentative diagnoses, patients who had an outpatient clinic visit under the diagnosis of hypertension less than twice would be excluded. Additionally, other data available in the NHIRD such as the date of birth, dates of visits, sex, type of admission or outpatient clinic visit, diagnosis, and treatment codes were also collected. Individual information is protected using scrambled identification numbers to prevent ethical violations related to the data. Patients older than 18 years were excluded. Our study conformed to the *Declaration of Helsinki* and relevant guidelines. The Institutional Review Board of Taipei Medical University approved this study (IRB no. N201911023) and waived the need for individual written informed consent.

### Metabolic syndrome-related diseases measurement

During the study period between January 1, 2000, and December 31, 2013, all identified hypertensive children and adolescents were evaluated for the presence of metabolic syndrome-related diseases based on ICD-9-CM codes, including DM (250.xx), hyperlipidemia (272.xx), and obesity (278.0, 278.1). For each hypertensive children and adolescents, one sex-, age-, and index year-matched participants without hypertension were randomly identified from the same database to form the non-hypertension control group.

### Statistical analyses

All statistical analyses were performed using the SAS Statistical Software, vers. 9.4 (SAS Institute, Cary, NC, USA) and R Statistical Software, vers. 3.6.1 (R Foundation for Statistical Computing, Vienna, Austria; http://www.r-project.org). Continuous variables are presented as the mean ± standard deviation (SD) and were evaluated by a two-sided independent Student's *t*-test. Categorical data are presented as sample proportions and were evaluated by a Chi-squared test. Conditional logistic regression analysis was conducted to calculate the association of metabolic syndrome-related diseases between the hypertension and non-hypertension group; the results were presented as odds ratios (ORs) with 95% confidence intervals (CIs). Statistical significance was set at *p* < 0.05.

## Data Availability

The data analyzed in this study were from Taiwan’s NHIRD. It is a restricted database only accessible by formal application to the Health and Welfare Data Science Center of Taiwan (https://dep.mohw.gov.tw/DOS/np-2497-113.html)^21^.
